# Comparing traditional news and social media with stock price movements; which comes first, the news or the price change?

**DOI:** 10.1186/s40537-022-00591-6

**Published:** 2022-04-28

**Authors:** Stephen Smith, Anthony O’Hare

**Affiliations:** 1 Department of Mathematics and Statistics, University of Strathclyde, Glasgow, UK; 2 Division of Computing Science and Mathematics, University of Stirling, Stirling, UK

**Keywords:** Sentiment analysis, Stock market, Twitter

## Abstract

Twitter has been responsible for some major stock market news in the recent past, from rogue CEOs damaging their company to very active world leaders asking for brand boycotts, but despite its impact Twitter has still not been as impactful on markets as traditional news sources. In this paper we examine whether daily news sentiment of several companies and Twitter sentiment from their CEOs have an impact on their market performance and whether traditional news sources and Twitter activity of heads of government impact the benchmark indexes of major world economies over a period spanning the outbreak of the SAR-COV-2 pandemic. Our results indicate that there is very limited correlation between Twitter sentiment and price movements and that this does not change much when returns are taken relative to the market or when the market is calm or turbulent. There is almost no correlation under any circumstances between non-financial news sources and price movements, however there is some correlation between financial news sentiment and stock price movements. We also find this correlation gets stronger when returns are taken relative to the market. There are fewer companies correlated in both turbulent and calm economic times. There is no clear pattern to the direction and strength of the correlation, with some being strongly negatively correlated and others being strongly positively correlated, but in general the size of the correlation tends to indicate that price movement is driving sentiment, except in the turbulent economic times of the SARS-COV-2 pandemic in 2020.

## Introduction

Twitter is a social media and microblogging website launched in 2006 by Jack Dorsey, Noah Glass, Biz Stone and Evan Williams which allows users to post a message of up to 140 characters (doubled to 280 in 2017) to be sent to their ‘followers’. It rapidly grew into a significant source of news, with 100 million users by 2012 and 321 million by 2018. It is popular among world leaders and business people, providing potential investors with a direct link to the people they will be investing in and a potential treasure trove of data for researchers.

On the First of May 2020, Elon Musk sent the tweet: ‘Tesla stock price is too high imo’. This single tweet wiped out nearly $15 billion, or 12%, of Tesla Inc ($TSLA) market valuation [1], and it is notable that this was not even the first time the much-discussed CEOs twitter feed had caused investors headaches. An August 2018 tweet where the billionaire claimed he was ‘considering taking Tesla private at $420’ and had ‘Funding secured’ led to an explosion in the share price (followed by a government investigation, $40 million in penalties, and the removal of Musk from his role as chairman [2]).

Of course, the phenomenon of CEOs statements affecting the value of their company is not new, though has been exaggerated by the speed at which Twitter and other social media can disseminate information. Famously, in the UK in April 1991, the eponymous owner and CEO of the jewellery chain Ratner Group, Gerald Ratner, delivered a speech to the Institute of Directors in which he mocked the quality of his company’s glassware and earrings decreasing the value of the company by $\pounds$500 million resulting in the company changing its name and sacking Gerald Ratner.

It isn’t only high placed company officials that can cause damage to the stock market valuation of a company. The socialite Kylie Jenner famously tweeted her displeasure with the a recent snapchat update, and SNAP Inc lost $1.3 Billion in value overnight [3]. A hacked Associated Press news account tweeted the false news that President Barack Obama had been injured in an explosion at the White House, a claim that was denied within minutes of the tweet being sent, but momentarily wiped $136.5 Billion off the value of the Dow Jones Industrial Average and the S&P 500 indexes [4].

In 2017 an examination of the president Trump’s impact on ten publicly traded companies mentioned in tweets between November 2016 and January 2017, the time between his election and swearing in [5]. Using event study methods, they found that a positive content in the tweet elicited positive abnormal returns in the company, and negative content prompted negative abnormal returns. The effect lasted less than one day for the most part and that within 5 days, there was no significant effect. The tweets led to an increase in Google search activity around the company, as well as an increase in volume of trading. The authors theorised that the tweet led to small traders reacting to the president’s tweet and that the impact was short term, meaning larger traders would see the tweet but not consider it important.

Further analysis of President Trump’s tweets in which he mentioned 58 companies before and after his election as president showed no significant effect on the company share prices or market indices caused by his tweets [6], the exchange rate between the Russian rouble and US Dollar was affected by his tweets [6], and tweets with a strong negative sentiment had a negative impact on a company’s share price, but tweets with a positive sentiment had no such impact [6].

Early in Twitters history research into classifying mood in tweets and its stock market predicting potential had already begun. Machine learning techniques were also found to perform reasonably well at classifying sentiment in tweets [7] but concluded that the algorithms could be improved. A study of 9.7 million tweets from 2.7 million users over a ten-month period, using the Google-Profile of Mood States to categorise them into one of six moods (Calm, Alert, Sure, Vital, Kind and Happy) and the Opinion Finder tool which measured positive vs negative ‘mood’ and found that certain public twitter moods could increase accuracy of models designed to use traditional market metrics (price, income etc) to predict market movements on the DJIA [8]. Specifically, the presence of a calm twitter mood could increase the accuracy of such prediction models, with the increased accuracy lasting 2-6 days, but that other moods did not improve prediction, and in fact sometimes made the models less accurate. The study also found that OpinionFinder tool mood value had no correlation with the DJIA closing. Since this study, mood analysis has evolved to be more commonly known as sentiment analysis, and public awareness of the data driven potential of social media has increased dramatically. In 2012 a corpus of Tweets was used to successfully build a sentiment classifier that was able to determine positive, negative, and neutral sentiments [9].

Other works have sought to correlate stock prices with Twitter data [10–16] finding some correlation between stock prices and Twitter data but the accuracy depended on data set used. More sophisticated machine learning algorithms have been employed [17–19] but found that an improvement in predictions of stock price predictions was possible but was limited to using financial data in their analysis. However, [20] warns against using a simple polar positive-negative classifier for sentiment analysis using trained datasets. An unsupervised approach using social media to garnering emotional context from text was shown to outperform machine-based learning approaches in the majority of cases [21]. Research has shown that, during the pandemic, reviews by shareholders have affected traders behaviour and consequently, stock prices [22].

Other works have focused on aspects of Twitter’s impact such as a 2012 study that investigated the correlation between the number of tweets daily related to the S&P 500 listed companies and the performance of stock indicators such as price and traded volume [10]. The authors found that the number of tweets mentioning S&P 500 stocks was significantly correlated with the daily closing price, the daily price change, and the daily absolute price change. They concluded again that at the stock market level, a model designed to predict whether the market will rise or fall using traditional metrics can be made more accurate by considering Twitter data.

A 2014 event study (a method of studying the the impact of an event such as a merger or CEO resignation etc.) applied sentiment analysis to a dataset of 400,000 tweets about S&P 500 companies [23]. The researchers distinguished between good and bad news, and found that returns were more pronounced before good news events than before bad news and that the impact of news events on the market differed largely across different categories. An adaption of this method using tweets gathered over 15 months from S&P companies but focusing on event studies over shorter time periods [13] found there was no significant correlation between prices and sentiment over the entire time period but found a significant dependence between Twitter’s sentiment and abnormal returns when Twitter is at its most active. It was found that this happened not only during expected twitter peaks (such as an earnings report or a major scandal) but also existed when the high twitter volume existed for less obvious reasons. The effect was small, less than 2%, but could last for several days, and interesting result due to its similarity to the similar result in the 2010 study.

Analysing non-traditional media sites for news that has the potential to influence a stock market evaluation of a company is a current topic of research. Investors are generally not keen to share the data used in their models so as not to give away any potential advantage, but some information on investor usage of Twitter is available. JP Morgan Chase & Co have talked publicly about their Volfefe Index. The Volfefe index claims that the market reacts to tweets containing certain key words, such as ‘China’, ‘Billion’ and ‘Democrats’. JP Morgan looked at Treasury yields and found in the minutes after a key tweet, the index can account for a significant number of the volatility in derivatives based upon the interest rates [24].

The Federal Reserve Bank of San Francisco published a 2017 paper which examined the most state-of-the-art approaches to sentiment analysis of economic data [25]. They examined a huge dataset of financial information from January 1980 to April 2015 containing thousands of articles form financial newspaper articles and their sentiment data in the form of a time series and compared the accuracy of established sentiment analysis tools by using pre examined articles and comparing the analysis results to human readers. They built their own model using their own vocabulary combined with economy-based sentiment analysis models and found a custom-built financial model performed better for analysing financial news than any ‘off the shelf’ sentiment analysis, drawing two conclusions from their dataset; firstly they found that news sentiment could predict consumer sentiment found in surveys, and that positive sentiment shocks resulted in increased consumer consumption, economic output, interest rates and would slow inflation [25].

The IMF also applied sentiment analysis to news articles from 20 countries between 1980 and 2019, assigning positive or negative sentiment to these items. They found that some issues in the analysis, such as evolving language over time meaning sentiment analysis needs to be adapted to remain accurate, but this did not affect results much [26]. For example, they found that news sentiment seemed to spike up before periods of economic depression, using Brazil in 1999 and Turkey in 2018 [26]. They also created a useful dataset containing thousands of financial news articles from all over the world that could be used for examining the difference in reaction to global shocks across borders or the effect that sentiment has on all types of markets such as currency exchange or interest rates or any other market [26].

Using traditional news, an analysis of the correlation between sentiment and stock price of 2 companies over a 10 year period using machine learning algorithms showed some positive results [27] but the authors noted improvements can be made. Specifically using news from financial based news organisations a high correlation between sentiment and stock prices volatility has been discovered [28, 29].

In this paper we analyse and compare the sentiment of traditional and non-traditional (social media) news sources relating to large companies and economic indexes using natural language processing. Our aim is to determine whether analysis of sentiment data can predict stock price movement and abnormal returns or if stock price predicts news sentiment. A major shock to world trade and markets in the form of the SARS-COV-2 pandemic occurred during this research so we include approximately an equal period of time before and after the outbreak of the pandemic and its subsequent shock, this limited the time period of our analysis but allows us to compare results to similar size studies [8, 13] that didn’t include such a shock. A benefit of taking a small time period is that we can ignore changes to language that indicate sentiment that challenging longer lived studies, e.g. [25, 26].

There are five questions being researched in this paper

1. Is there any correlation between price movements in the stock market and the sentiment of tweets and traditional news? To answer this question market movements and sentiment data will be compared by gathering time series data for both, then use Pearson’s correlation coefficient to quantify the relationship.
2. Is there is a correlation between the Tweets of World leaders, news stories about their countries and the economic performance of a country, represented by benchmark share indexes in each nation. This will be assessed in a similar way to the first question, using Pearson’s correlation coefficient and t-test to examine whether a relationship exists.
3. Can sentiment data be used to predict a stock’s performance relative to the market? To examine this each share price movement will be compared to the movements of the key economic index, the S&P 500, by subtracting the daily percentage change in the S&P from the percentage daily changes in the individual stocks price. This will provide a value of for how the stock is performing relative to the market each day, which can then be compared to the sentiment data using Pearson’s Correlation coefficient.
4. Is news coverage and Twitter sentiment driving stock market prices (both absolutely and relative to the market) or is stock market movement driving news coverage sentiment. This will be assessed using Time lagged cross correlation which shifts one time series incrementally and measures the correlation each time. The time series of price movement will be shifted from minus five days to plus five days, so will return 11 correlation values, one for the correlation between prices and the sentiment five days later, one for the price changes and sentiment four days late, and so forth. If the highest correlation is at the centre then that indicates the two are most synchronised at the same day, but if the correlation peaks earlier in one of the time series then that would indicate that one series is leading the other.
5. Are the results similar in troubled economic times and in normal times. The way this will be judged is by dividing the datasets in two, comparing results in data gathered in 2019 with results in 2020 since 2020 brought the COVID-19 pandemic crisis and resulted in some historically unprecedented challenging market conditions, providing an ideal opportunity to repeat and compare results in smooth market conditions and turbulent times.

## Methods and data

Opening, closing and volume data is easily available online, but second by second trading data is not as well recorded. The accessibility of daily data lends itself towards examining daily returns and the nature of news makes it easier to examine its impact over a full day rather than moment by moment.

### Choice of companies

In this paper we will analyse data gathered from the 1st of January 2019 until the 15th of July 2020 to cover an equal period before and during the global SARS-COV-2 pandemic. We decide on which companies to analyse based on the following criteria:

1. The company must be publicly listed so the valuation can be tracked.
2. It must also be from English speaking countries as the sentiment analysis tools used here are designed for the English language.
3. It must also have a CEO with an active Twitter feed.
4. The Twitter account must be verified by Twitter, but some exceptions were made for accounts that were unverified if they were tagged (linked to by another account by their Twitter username specifically) by the official verified Twitter account run by the company in question. The Twitter account also needed to have enough tweets, so any account with fewer than 100 tweets was dismissed. After applying these rules, twenty-three accounts remained.

Most executives do not use Twitter, and many who do, do not use it regularly enough to give enough data to examine the effect. To find enough companies to analyse we decided to look at the largest companies on the S&P 500 Index. We looked a the 200 companies (by market capitalisation) in the S&P 500 list and 23 companies satisfied our requirements (see Table 1).

**Table 1:** Selected Stocks and Twitter Representatives used in our analysis

Company	CEO
Microsoft	Satya Nadella
Alphabet (Google)	Sundar Pichai
Apple	Tim Cook
Disney	Bob Iger
Verizon	Hans Vestberg
Cisco	Chuck Robbins
Salesforce	Marc Benioff
Accenture	Julie Sweet
Medtronic	Geoff Martha/ Omar Ishrak
Twitter	Jack Dorsey
Starbucks	Kevin Johnson
Intuit	Sasan Goodrazi
ServiceNow	Bill McDermott
AMD	Lisa Su
Equinix	Charles Meyers
Activision	Bobby Kotick
T-Mobile	Mike Sievert/John Legere
Southern Company	Tom Fanning
Illumina	Francis deSouza
Fiserv	Jeff Yabuki
Autodesk	Andrew Anagnost
Humana	Bruce Broussard
Waste Management	Jim Fish

The criteria for selecting these are that all Companies/CEOs are in the largest 200 companies in the S&P 500, have tweeted at least 100 times since January 2019 and are verified or have been mentioned by verified company account. The list excludes Amazon and Jeff Bezos for reasons explained in the text

These were the CEO of their companies on January 1 with the following exceptions; Sundar Pichai has been the CEO of Google since 2015, but Google is a subsidiary of Alphabet Inc, and Alphabet is the company listed publicly on the stock exchange. Sundar Pichai is included because he was the public face of the company even before he was appointed the CEO of Alphabet in December 2019 and Google is the flagship brand of Alphabet Inc, owning an running most of Alphabets most famous and profitable products (such as YouTube and Android) so Sundar Pichai is an acceptable public representative of Alphabet over the entire period. Bob Iger of Disney stepped down as CEO on the 25th of February to be replaced by Bob Chapek. However, Iger remained at the company in a senior-role as Executive chairman, but due to the coronavirus pandemic Iger returned to his previous role to help navigate the crisis. Julie Sweet of Accenture, was the CEO of Accenture’s North American business and features on Forbes most powerful woman list from 2016 through 2019 before taking over as CEO in September 2019 and is included in our list due to her high public profile and the fact the previous CEO had no Twitter presence. T-Mobile replaced John Legere with Mike Sievert on the 1st of April 2020 and since both men had an acceptable Twitter presence our dataset uses John Legere’s Twitter sentiment until the day of the changeover, then uses Mike Sieverts Twitter activity for the rest of the period. The same method was used for Omar Ishrak and Geoffrey Martha of Medtronic, with Martha replacing Ishrak in April 2020.

**Table 2:** The countries, their elected leaders and benchmark Indexes used in our analysis

Country	Leader	Index
UK	Boris Johnson	FTSE 100
Canada	Justin Trudeau	S&P/TSX
Australia	Scott Morrison	S&P/ASX 200
Ireland	Leo Varadkar	ISEQ
New Zealand	Jacinda Ardern	S&P/NZX

### Choice of political leaders to include

We also included world leaders Twitter accounts to investigate the link between heads of state and national indexes. President Trump’s tweets attract so much attention already, so five other major economies where English is the primary language were chosen and the tweets of their heads of state were compared to the share price index movements, listed in Table 2. Boris Johnson became prime minister of the UK in late 2019, so has fewer data points than most of the CEOs and world leaders. Likewise, Leo Varadkar was replaced as Taoiseach by Micheál Martin in June 2020, so has a few less data points than the others.

### Choice of traditional new sources

Historically, the best source of financial news has been newspapers, with the majority of breaking stories go online long before they appear in print media. We assumed that the news financiers are most likely to be influenced by are financial publications. The websites and papers of these organisations tend to feature punditry from experts in their field and given the number of articles required to draw any conclusions, separating punditry from breaking news was difficult. To counteract this, we will examined two different sources of news, one from a financial news website and one from a traditional news website.

We had one requirement here; that the website be free to access. Forbes is the largest global business media brand in the world and claim to reach more than 120 million people worldwide and was chosen due to its strong reputation for business news and its strong online presence. For the traditional news, the site needed to be based in the USA and have no particular focus on finance. The Associated Press based in New York City and has a reputation for being quick to report breaking news and being frequently used as the primary news source for other publications and is often republished by other news sources. To differentiate between the two sources, we will refer to AP News as News and Forbes as Punditry.

### Gathering data

We used Google’s built in tools to limit a search for news articles limited to individual websites (AP and Forbes), to only include results with a certain word in the title and to limit results to a certain time period. Amazon Inc. was removed from our data set as its name with the Amazon Rainforest which has been in the news frequently in the time period between January 1st, 2019 and July 15th 2020 due to ongoing forest fires (We attempted to distinguish the two sets of stories by removing articles where the titles contained certain key words, such as ‘Jair’, ‘Bolsonaro’, ‘Fire’, and ‘Burn’, but too many stories about the burnings made it through the filter). In the case where several news articles appeared on the same day we aggregated them into a single article for analysis.

To gather the tweets, a free service provided by vicinitas.io was used to download the last 3200 tweets for each CEO in our dataset [30]. Retweets were left in as it was believed retweets from high profile individuals can be newsworthy, such as when the US president retweets controversial political figures, so there is potential for market movement due to a retweet. We retained the the tweet text and the date it was sent from the CSV that is produced by Vicinitas where, similar to the news articles, all tweets from the same day were aggregated into a single tweet in preparation for sentiment analysis.

### Analysing sentiment

For the sentiment analysis we used the Python library, “Natural Language Tool Kit” or NLTK, which has a built-in sentiment analysis tool called VADER (“Valence Aware Dictionary and sEntiment Reasoner”). A words Valence is its goodness or badness and VADER uses a dictionary of words and sets of rules to decide each word valence using what is referred to as a bag of words model. The bag of words model used here simply applies a value to each word and decides the valence based on the ratio of positive to negative words. There are machine learning models that can account for context by examining the words surrounding each word and looking for patterns that indicate other meanings. These are useful in theory but often require training for use on specific datasets.

VADER was chosen for two reasons, first it is fast and requires no training, for the sake of this research training might improve the accuracy but time constraints mean this is not practical. Another major advantage of VADER is that it is trained on social media data so is useful for analysing Twitter data, ideal for our gathered tweets. Vader returns a dictionary with four values, “positive sentiment” (the sum of the value of positive words i.e. great, brilliant), “neutral sentiment” (sum of values of neutral words i.e. he, them), “negative sentiment” (sum of value of negative words i.e. poor, rubbish) and “compound sentiment” (ratio of positive to negative words). The compound score returns a value between $-1$ and 1, with $-1$ being very negative and 1 being very positive. The compound score best meets the needs for the analysis so it is used going forward.

The News, Pundit and Tweets data were all run through the VADER package and the compound scores for each day were extracted. This returned a data frame for each company and country with dates and three scores, one for the News, Punditry and Tweets.

### Calculation of correlations

We use the Pearson correlation coefficient to calculate the correlation between two variables. This gives a value between -1 and +1 that measure the linear correlation between two variables X and Y, made up of n values of x and y. The formula for Pearson correlation coefficient is

1 $$\begin{aligned} \rho _{x,y} = \frac{cov(X,Y)}{\sigma _x\sigma _y} = \frac{n\sum xy - \left( \sum x\right) \left( \sum y\right) }{\sqrt{\left( n\sum x^2 - \left( \sum x\right) ^2\right) \left( n\sum y^2 - \left( \sum y\right) ^2\right) }}, \end{aligned}$$

where $\sigma$ is the standard deviation (in x and y), $\rho$ is known as the population correlation coefficient, in the current research that would be the correlation coefficient of all financial news articles and all stock price movements which is obviously not possible.

In statistical analysis, we rarely have data on the whole population so the correlation calculated is the sample correlation coefficient, denoted by *R*. We use the ‘cor.text’ function in ‘R-studio’ to calculate the correlations where three values are returned, an *R* value, a p-value and a confidence interval. From the sample we have we attempt to calculate the population correlation, more specifically whether the population correlation coefficient is different from 0. To do so, we perform a hypothesis test with the following null and alternate hypothesis

*Null Hypothesis: H*$_0$: *Population correlation coefficient is equal 0* ($\rho = 0$)

*Alternate Hypothesis: H*$_1$: *Population correlation coefficient is not equal 0 *($\rho \ne 0$)

We perform the hypothesis test at at the 95% significance level, meaning we reject $\hbox {H}_0$ in favour of $\hbox {H}_1$ if p $<0.05$, and do not reject $\hbox {H}_0$ if p $>0.05$. Since we know the number of data points in the sample and the sample R, we can test the null and alternate hypothesis using the following test statistic.

2 $$\begin{aligned} t= \frac{r\sqrt{n-2}}{\sqrt{1-r^2}}. \end{aligned}$$

This $t-$value is then examined against statistical tables to get a p-value. We will concentrate our results on the calculated p-value. If p is greater than 0.05 then there is insufficient evidence that the population mean is not equal 0, so we cannot say there is any correlation. If p $<0.05$ then we can report the sample correlation and examine the direction and strength.

To test the direction of the correlation (if any) between variables we use time lagged cross correlation is a which shifts one time series incrementally and measures the correlation each time. This will demonstrate whether the news or tweets precede or follow movements in stock prices. The time series of price movement will be shifted from minus five days to plus five days, so will return 11 correlation values, one for the correlation between prices and the sentiment five days later, one for the price changes and sentiment four days late, and so forth. If the highest correlation is at the centre then that indicates the two are most synchronised at the same day, but if the correlation peaks earlier in one of the time series then that would indicate that one series is leading the other.

## Results

Since the data is gathered from the top of the S&P 500, and the index is weighted by market capitalisation, it is fair to assume that these shares are a reasonable picture of the market. It is perhaps not surprising that the CEO of Twitter himself, Jack Dorsey, is the most active over the time period. It is however interesting that the second and third most prolific tweeters, Hans Vestberg and Chuck Robbins, both run the only two telecommunications companies on the list, Verizon and Cisco respectively. We first took the summary data of the number and sentiment of stories published by both news sources and analysed them to see whether the two are correlated, to examine whether their news priorities are the same and whether the same stories are reported in the same tone.

**Fig. 1:**
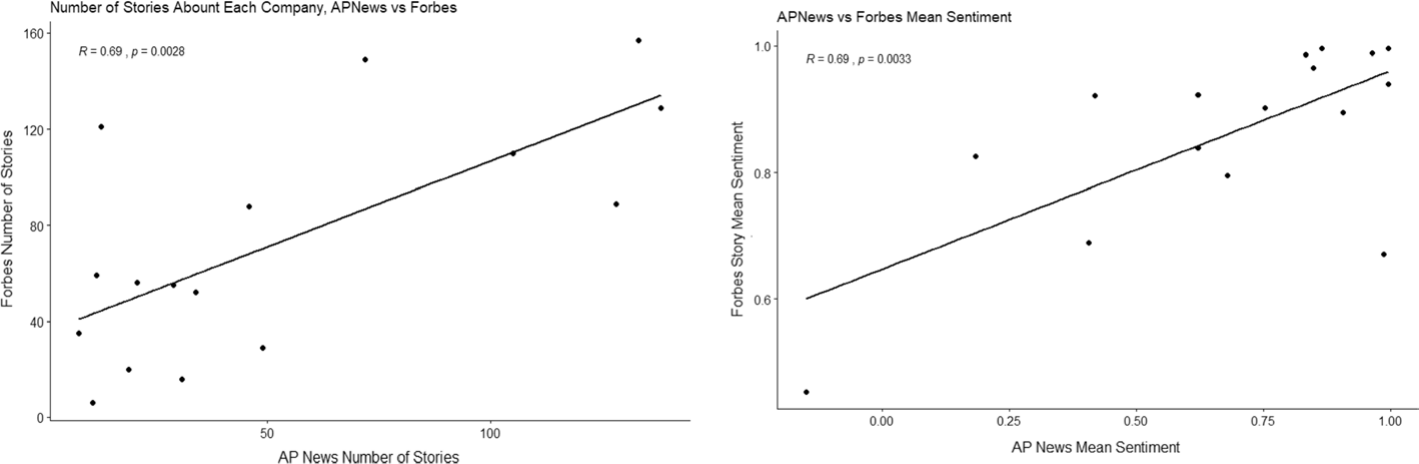
Number of stories published by AP News and Forbes (left) and their mean sentiment (right), about each company between January 2019 and July 2020 with regression line

### Sentiment and frequency of news items, tweets

The number and sentiment of stories published by both news sources in our data set (Forbes and AP News), Fig 1, were analysed and we found a positive correlation between the sentiments ($R=0.69$, $p=0.0033$) and frequency ($R=0.69$, $p=0.0028$) of the two publications so we conclude that Forbes and AP News do report roughly the same stories in the same way.

**Fig. 2:**
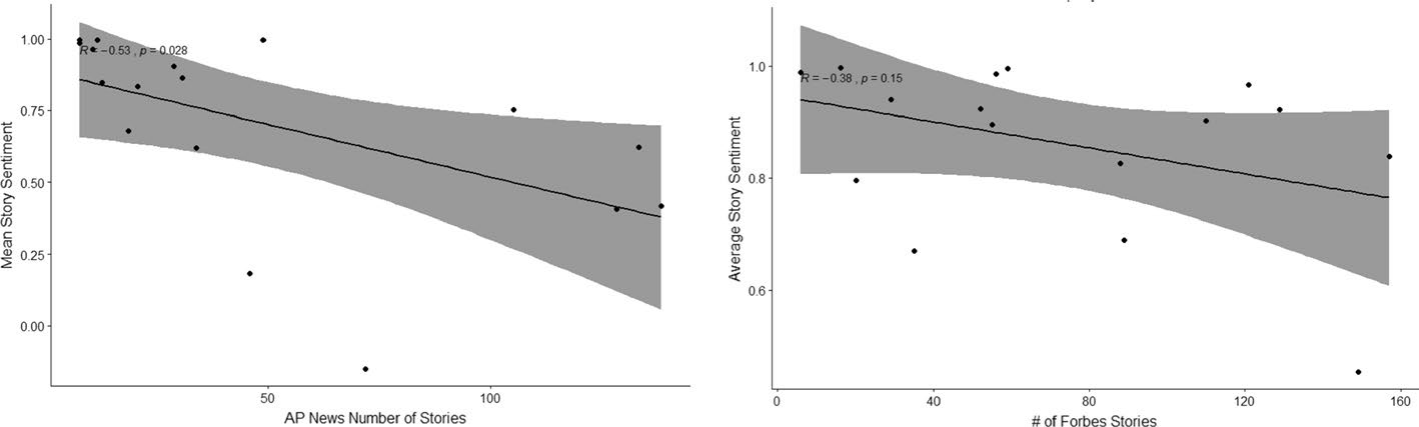
Mean number of stories published by AP News(left) and Forbes (right), vs the average sentiment of those stories

**Table 3:** Correlation between News/CEO Twitter Sentiment and Daily Company % Price Movement

Company	Twitter p (r=0)	AP News p (r = 0)	Forbes p ( r= 0)
Accenture	0.8338	0.6956	0.03442
Activision	0.6011	0.2365	0.008021
AMD	0.6377	0.3242	0.7414
Apple	0.48	0.2529	0.6946
Autodesk	0.2202	N/A	N/A
Cisco	0.03662	0.6821	0.6183
Disney	0.3327	0.4325	0.1276
Equinix	0.4516	N/A	N/A
Google	0.7602	0.2813	0.5341
Illumina	0.2104	N/A	N/A
Intuit	0.05836	0.5349	0.9512
Medtronic	0.004417	0.9541	0.00499
Microsoft	0.9298	0.83814	<0.0001
Salesforce	0.3149	0.6389	0.3799
Service-now	0.4996	0.6654	0.4579
Southern Company	0.4735	0.9589	N/A
Starbucks	0.8551	0.06357	0.7678
T-Mobile	0.4474	0.8225	0.7723
Twitter	0.4673	0.538	0.1329
Verizon	0.5091	0.04342	0.07269

We only note six significant correlations (Cisco (R = $-0.14$), Medtronic(R = $-0.408$), Accenture (R = 0.39), Activision (R = $-0.44$), Medtronic (R = $-0.602$), Microsoft(R = 0.37)), 4 of which are negative

**Fig. 3:**
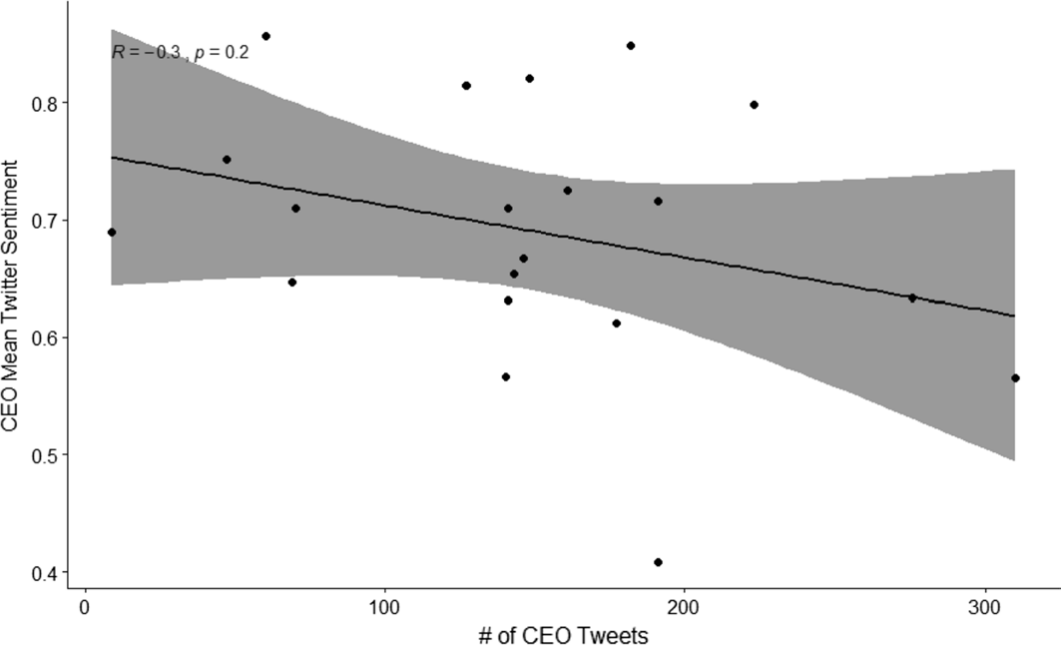
Number of Tweets for each companies CEO in the time period vs their average sentiment score

The business magazine has no significant correlation between number of published stories and their sentiment ($R=-0.38$, $p=0.15$), while AP News has a negative linear relationship between the two ($R=-0.53$, $p=0.028$), Fig. 2, meaning the more stories about a company AP News publishes, the worse the average sentiment is. AP News primarily deal with breaking news stories, so it is possible that the reason for more frequent news about a company is bad news, confirming the old adage “no news is good news”. A scatterplot of the the number of tweets by a CEO and their mean sentiment, Fig. 3, didn’t show any significant correlation ($R=-0.3$, $p=0.2$).

**Table 4:** Correlations between world leaders tweets/news articles and key national indexes

Countries	Twitter p (r=0)	AP News p (r = 0)	Forbes p (r = 0)
UK	0.06898	0.3775	0.8945
Ireland	0.714	0.6275	< 0.0001
Canada	0.8328	0.2067	0.8683
NZ	N/A	0.5381	0.8562
Australia	0.5289	0.7957	0.6951

The only significant correlation is between Ireland and Forbes, with an R value of 0.6388, a very strong correlation

**Fig. 4:**
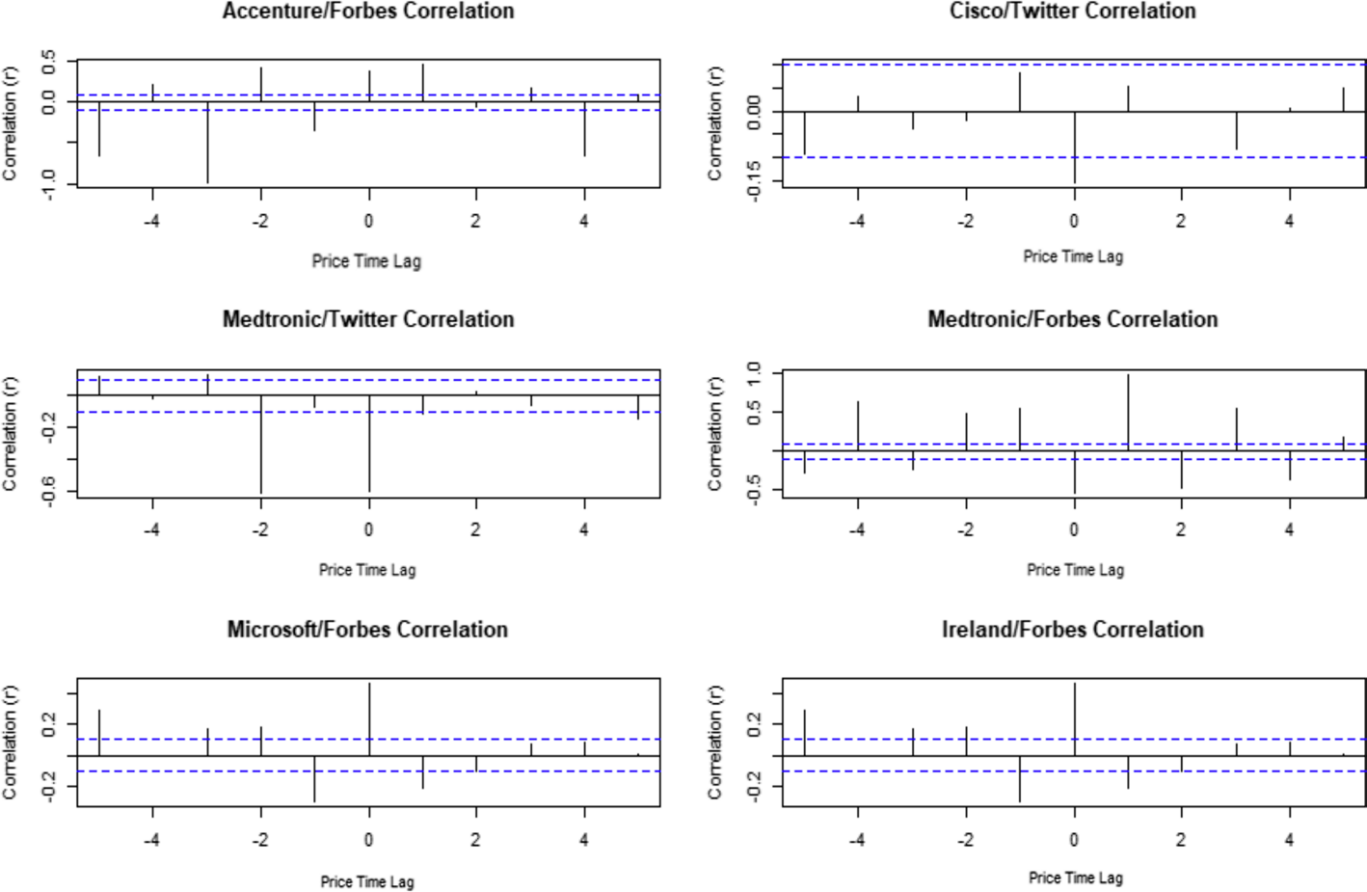
Time Lagged Cross Correlation for companies where population correlation is not equal 0 (Actual Returns). The plots show the values of the correlations between the movement and sentiment for up to 5 days either side of the publication of the news. There is no clear pattern to these results

**Fig. 5:**
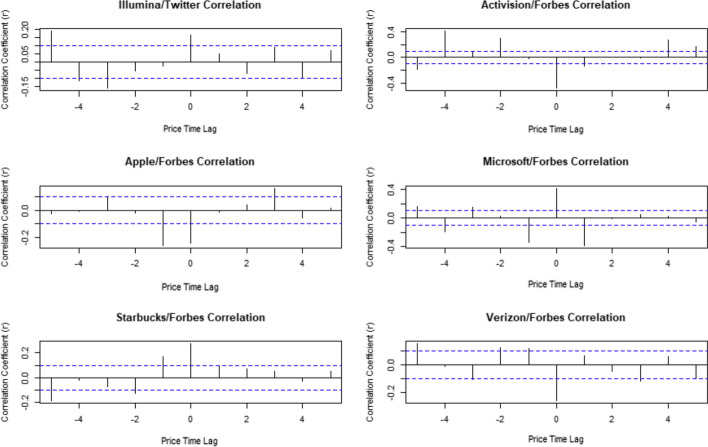
Time Lagged Cross Correlation for companies where population correlation is not equal 0 (Relative Returns). The plots show the values of the correlations between the movement and sentiment for up to 5 days either side of the publication of the news. Again, we see little evidence of a pattern

We finally tested whether the mean sentiment data for each company is related to the minimum, mean and maximum movement for each company and whether the number of tweets and articles over the entire period affected the movements and found two significant relationships between the worst day of trading for each company and the average sentiment of both financial and regular news. The average news sentiment for each company from each source is strongly positively correlated with the percentage price movement on their worst day of trading ($R=0.59$, $p=0.013$). It is unclear what might cause this effect, it is possible that the bad news put out by these sources are what causes the days which a share price is hit worst, or perhaps the collapse in stock price causes a flurry of negative press resulting in a significant worsening of average sentiment (Figs. 4, 5).

### Sentiment correlation with stock prices

Calculating the correlation for each company and performing a t-test to determine whether the daily stock price change was correlated with the sentiment on each day showed there was no correlation between the non-financial (AP) news and the daily price movement. Twitter showed a small negative correlation for Medtronic ($R=-0.408$) and Cisco ($R=-0.14$) and Forbes magazine showed a larger correlation but the direction was not so clear (Accenture, $R=0.39$, Activision, $R=-0.44$, Medtronic, $R=-0.602$, and Microsoft, $R=0.37$). Since correlation does not indicate causation so we can only speculate as to the reason. We didn’t look at price volatility and a longer study that showed financial based news items had a strong correlation with stock price volatility [28, 29]. In turbulent times, when volatility is high it has been observed that public mood does drive changes in stock prices [22].

There are some connections between the four companies correlated with Forbes, Table 3. Medtronic and Accenture are in different industry sectors, but both are headquartered in Dublin Ireland, although this is primarily for tax reasons as both are most active in North America. Microsoft and Activision are both major presences in the video gaming industry, and it might be the case that the video game industry is sensitive to news as its revenue is entirely dependent on public satisfaction with their products, but Microsoft’s “Xbox” gaming only accounts for 10% of its revenue and the correlation is strongly negative. There is no clear link between Cisco and Medtronic, both are in entirely different industries and neither has CEOs who are a household name, so once again there is no clear pattern.

Given the connection between Medtronic and Accenture we asked whether economic performance is correlated with the news and with the Twitter activity of the head of government and we found no significant correlation for the United Kingdom, Canada, New Zealand, and Australia in Twitter, AP news, or Forbes, Table 4. We did, however, find a significant correlation between Ireland and Forbes, with an R value of 0.6388, indicating a very strong correlation. This looks interesting, especially considering the significant correlation found between the two companies headquartered in Ireland and Forbes. Forbes is a USA based publication and any significant news about the Irish economy is likely to break first in Ireland. Ireland’s low business tax rate and European Union market access makes it an attractive prospect for most businesses from outside Europe to headquarter their European business. Forbes is likely primarily interested in the news from Ireland that would affect such businesses based in the US, and this is a possible reason why business reporting might have a stronger relationship with Ireland than the other countries. It is likely that for a story to be of interest to Forbes, it would have to affect businesses significantly enough for the market to react, and Forbes would likely not take an interest until the effects of the story (such as price movements) have been felt. This is speculation however as there is not enough information to determine the cause.

### Time-lagged correlation

When we look at the time lagged cross correlations for those companies that showed some correlation between price change and news we saw no clear pattern. We did see some hints that when the news predates the change in stock price there are higher correlation coefficients than when the stock price change comes first, suggesting that the sentiment of the media coverage and Twitter activity is driving changes in stock price rather than the price driving the coverage.

It is possible that there is time lagged correlation for the companies that are not correlated. Checking more companies for time lagged cross-correlation is a promising area for future research.

2019 was a fairly stable time for the stock market. There was no major election in the United States and the Covid-19 crisis had not begun for most of the world. This is reflected in the S&P 500 by the fact the largest monthly rise and fall were 6.89% and 6.58% respectively. This is in contrast to 2020 where the COVID-19 crisis has led to turbulent economic times with mass layoffs and government bailouts in most major economies where the largest monthly rise and fall were 12.68% and 12.51% in the indexes value.

We now examine whether this economic turbulence affects our analysis. The dataset for the entire time period contained 386 trading days; 252 days in 2019 and 134 in 2020. For 2019, only Activision has a significant correlation between the daily price change and the news sentiment on that day. We also see the first significant relationship for non-financial news, between Verizon and AP News. Twitter activity has no significant effect on anyone suggesting that in normal times Sentiment analysis of Twitter may not be of any use.

The 2019 relative returns show only a significant result for Verizon but across all sources (Twitter, AP news, and technical news articles). Verizon was the worst performing company on average over the period we looked at and this may make investors uncomfortable and reactionary to any outside influence. Again, there was no clear pattern in time lagged correlations. Correlations are most significant on the day of publication, suggesting the impact of the news is short lived, but there is some sign of correlation before and after publication.

We found more correlations in 2020 between the daily percentage price movement vs sentiment on that day. Once again there is no clear reason for the relationship, but with one fifth of the chosen companies being predictable with Forbes, it is fair to say there is probably a use for financial news in prediction. It would also suggest the markets are more interested in the news during periods of high volatility which would make sense, if normal models are not working due to uncertainty then the market may get more reactionary.

A previous study has shown some correlation between stock price volatility and public mood on social media [22] which corresponds to our findings in 2020. The fact that we don’t see this in 2019 is interesting.

There is no clear pattern to the time lagged correlations. Although the pattern is inconsistent is notable that the left half of the plots all have higher correlation coefficients than the right. This would suggest that although we cannot see a precise effect, it is more likely the price is driving the sentiment of the media coverage and Twitter activity rather than the coverage driving the change in stock price.

The relative returns in 2020 showed no improvement compared to the absolute returns. This would suggest that in times of upheaval it would be better to examine the absolute returns over the relative returns. The time-lagged correlations did show some evidence of price movement driving sentiment, but the opposite was not there before. This implies the financial news can be used to help predict movement in financially troubled times, but Twitter struggles to predict even then. There is limited evidence of market movements driving CEO Twitter sentiment, but this is not much use for investors. There is still no clear pattern with the positivity and negativity of the correlations on day of publish or before/after.

The relative 2020 returns show evidence of share performance predicting the CEOs Twitter sentiment, but no evidence of the opposite. A study over a similar time period but analysing posts from twitter users (not just CEOs) showed no strong correlation between social media sentiment and stock prices movements [13]. Two of the time-lagged correlation results show evidence of financial news predicting share performance, as the magnitude of the right-hand side is significant, but neither is particularly strong. It is still promising that there is some evidence of sentiment driving prices, meaning this may be a good thing to research further.

## Discussion

The original research question was whether the sentiment data gathered could be used to predict stock price movement, and it appears in some cases it can, but the results are not that clear cut. The easiest question to answer is whether the national economies, represented by benchmark national indexes, are correlated with Twitter and news stories and to this we can say in almost all cases they are not. None of the heads of government tweets were significantly correlated and there are some simple reasons this may be. World leaders are first and foremost politicians and their Twitter accounts are almost certain to be run, at least partially, by campaign staff and advisors. They are not likely to be of much interest to most investors as actual major policy announcements tend to come from other places. As for the failure of news in this work, there is another simple explanation; the sources used. This study was focused on the US economy so used American news websites and did not consider local reporting or time differences. As an example, there is a 16 hour time difference between New Zealand and New York, meaning news published about the New Zealand economy from a New York source would have to be published between 5.30 pm and 12.30 am to arrive when the market is open. Although news is 24-hours, punditry and serious analysis is not likely to be published at such odd times, meaning they are unlikely to be noticed by New Zealand investors.

For future research into predicting national indexes there are some areas that could be investigated. The first is choosing a wider range of Twitter accounts to analyse, by considering other cabinet members or multiple government related accounts it might be possible to build a more complete picture of the country’s leader’s impact upon the economy. It might also be worth considering whether the trustworthiness and approval ratings of leaders affects their impact upon the market. By examining approval polls and electoral success of leaders, it could be established whether a highly popular leader such as Jacinda Arden impacts the economy more than a more divisive leader such as Boris Johnson. It could also be examined whether political leaning affect leader’s impact on market one way or another. Another area to look into is whether correlation is found using local news sources with more accurate publish dates, and if investors are more likely to watch local sources perhaps that would be more impactful in their behaviour than foreign news.

The main research question was whether price movements were dependent upon Twitter and news sentiment. It is important to note there was almost no significant correlation with regular news sentiment, so any further research should probably focus on financial news sources. As far as Twitter feed’s usefulness, there were four CEOs whose tweets sentiment were found to be correlated on various occasions. Geoffrey Martha and Omar Ishrak of Medtronic and Chuck Robbins of Cisco tweets sentiment were correlated with the overall actual returns of their company and with the actual returns during the 2020 COVID-19 crisis. Francis deSouza of Illumina’s tweets were correlated with the relative returns of the market while Hans Vestberg of Verizons tweets were correlated with the relative returns in 2019. This is not a bad result, but it is interesting that most of the significant Twitter correlations are negatively correlated, suggesting perhaps that investors react poorly to CEO tweets, or possibly that executives feel the need to speak out when their stock is performing poorly. The time lagged cross-correlation is inconsistent in its direction, but the strength of signals would certainly suggest that the price movements are driving the Twitter activity.

There are a couple of key areas to examine to look into this further, the first is time scales. As previously discussed, the data gathered here focussed on daily data, but what if the data was used on a smaller or larger time scale? Do these CEOs have an intra-day impact with their statements which cannot be seen within daily returns? Minute by minute data is harder to find but is available, and it could be examined whether each trade can be predicted using the Tweet sentiment immediately preceding it? There is some evidence here that over a longer period there is more correlation between sentiment and price movements, and its possible this could be true over even longer periods. Is it possible average sentiment over time could be used as an indicator for abandoning an investment for position traders if sentiment indicates problems ahead.

Another potential area to improve is changing the relative returns method. It may be better for any future examination to look at more sophisticated methods of expected returns such as using the popular CAPM model and calculating returns relative to those results or using any other method of stock price prediction. If a researcher had access to a sophisticated model belonging to an investment firm or bank, the sentiment data could be assessed with actual returns relative to the model predictions. This is not limited to the Twitter data, all correlation could be checked relative to any prediction method to examine whether sentiment can improve its accuracy.

The last potential area of interest that could be examined is, why are these particular CEOs different to the other listed. These were only the appropriate executives from the 200 largest S&P 500 companies and there may be many more out there who are and are not significant. By gathering a large list of CEOs you could examine the difference be- tween those who impact their stock prices and those who don’t. It could be due to the frequency of their tweets, the performance of their company, the investor confidence in CEO leadership or many other reasons. Finding the missing link between the CEOs Twitter activity and the price movements could be an excellent way to find value in the market others do not see.

Financial news was the most successful source of correlation overall, with more than one in five companies price movement being significantly correlated with financial news sentiment. They ranged from famous companies like Microsoft to more obscure companies like Southern Company. Similar to Twitter it would be worth examining different time-scales, but there are other things to look at as well. This paper focused on a single source of financial news and although Forbes is a very influential publication, there are many more publications that are just as prestigious as Forbes. By scraping articles from the same day from more publications a researcher could examine the impact of each individual story on prices or could combine all news into one and examine the effect of the collective of news.

Time lagged cross correlation had no real pattern of positive or negative correlation, but in general the strength of the correlation suggested price impacts sentiment, with one key exception: the 2020 relative returns vs Twitter and Forbes. It is not particularly strong, and there is no pattern to the number of days, but there is some evidence, and this presents itself as an opportunity for further research. The economic impact of the 2020 coronavirus crisis is ongoing in most countries, and with more data it could be made clear whether this phenomenon continues or even gets stronger. Data could also be gathered from the 2008 financial crisis, although this may not be able to assess the impact of Twitter as Twitter was in its infancy at the time, or during local economic shocks like the 2016 EU Referendum in the UK. It may be that sentiment analysis is most useful in such troubled economic times as investors get nervous and anticipate bad news.

There are of course other methods that could be used to assess sentiment impact, but the research here indicates there is some potential use. More research is required over more timescales, and the exact nature of the relationship is not clear yet, but the potential is there for executives Twitter accounts and financial news to help predict price movement.

## Conclusion

We have compared traditional news from reputable outlets, tweets from CEOs of some major companies and world leaders with stock prices and indices over a period that spanned the outbreak of covid-19 worldwide pandemic. The aim of this study was to investigate the feasibility of using different news items for predicting stock prices movements (or if the price preceded the news).

Our results show some evidence of financial news predicting stock price and, generally, the opposite for CEO posts, but neither is particularly strong. We only found one instance of significant correlation between sentiment a countries index but this may be explained by the news outlet only reporting significant news so there is an element of censorship or filtering those stories that would do not correlate but it is still promising that there is some evidence of sentiment driving prices, meaning this may be a good thing to research further. Correlations between sentiment and price movements, where they exist, are most significant on the day of publication, suggesting the impact of the news is short lived. We found more correlations in the pandemic period than the pre-pandemic period and we can find no clear reason for this. There was some small evidence that the price movements were driving sentiment from time-lagged correlations, but the opposite was not there before the onset of the covid-19 pandemic. This implies the financial news may be used to help predict movement in financially troubled times, but Twitter struggles to predict even then.

Certainly, negative tweets by a leading politician or CEO can decrease value in a company but due to the infrequent nature of such events we didn’t see any in our rather limited time period. A larger study (going back further in time) here would be skewed by the effects of the global pandemic that broke out during the data gathering phase of this research and in evolution o1f the language that signifies sentiment so we limited our period so that we included an equal amount of time before and after the outbreak. In the future we would like to see the time period extended so these rarer events would be included in the analysis and machine/deep learning algorithms employed to process the feelings of a wider population of social media users.

## Data Availability

All data used in this analysis is freely available on the internet and detailed instructions on how we obtained and analysed the data are given in the text.
